# There is a Place for Passion

**DOI:** 10.15694/mep.2017.000213

**Published:** 2017-11-28

**Authors:** Itoro Udo

**Affiliations:** 1 St. John's Unit

**Keywords:** international medical graduates, near-peer teaching, access, medical education, culture

## Abstract

This article was migrated. The article was marked as recommended.

Passion generates followership. Likewise low morale can be corrosive. This article reflects on the unique circumstances that lead this clinical educator to develop his interest in medical education. Nurture and exposure seem to have been crucial influences in generating interest. Tangible gains and positive educational outcomes sustained it. Some current challenges for clinical educators in the UK’s National Health Service are highlighted. These include pass rates for international doctors in postgraduate exams, gradual erosion of clinical teaching time and low morale among junior doctors.

## Early Exposure

I have often wondered about what it was that made me become passionate about medical education. MedEdPublish’s current theme of Accessing Medical Education has generated lots of personal reflection and discussions with people with whom I shared common experiences. This has led to this articulation of my views and opinions as an international clinical educator.

Most of us who grew up in St. Luke’s Hospital, Anua have gone into healthcare professions. St. Luke’s was then a renowned general hospital in South-eastern Nigeria ( Nolan. 1949; Hilton & Ward. 1998). Founded in 1947 by the Irish based Medical Missionaries of Mary (MMM), ( Medical Missionaries of Mary. 2017) it served a population of at least four million and its reach extended far. The unique features of St. Luke’s were that, for its time, it was very well staffed and well equipped. By the 1980s, both government and mission employees were working harmoniously side by side. For its time, it had consultants and subspecialists- anaesthesiologist, paediatric surgeon, radiologist, etc and abundant nursing subspecialists.

My parents worked there as physician and pharmacist. We, then lived within the grounds of the hospital, as did most of the other medical staff and their families. The staff quarters had lovely, expansive green landscape of rolling hills and trees which made fantastic playgrounds for us children. The quarters had the hospital and convent to its east side and the also renowned School of Nursing, Parish Church and Primary School to its west side. Behind us was a ravine and forestland.

Based on its founding, we were often woken by the church bells calling for morning prayers and we would see the nuns crossing the expansive landscape to and from prayers, into the hospital to begin the day’s work. This, they did day after day. Most of them, at the time were of Irish ancestry. Apart from the indigenous Nigerian doctors who were all first generation medical graduates in their communities and mostly UK trained, there were also international medical staff, mostly from Asian countries who also worked there. So it was a very international mix of staff and their children from White, Asian and Black backgrounds.

## Passionate Professionals

St. Luke’s was particularly renowned for its Obstetrics & Gynaecology department, headed by the much beloved Dr Ann Ward FRCOG, now of blessed memory ( Irish Times. 2016). At the time, it had some postgraduate training accreditation with the Royal College of Obstetrics & Gynaecology. It also had a steady stream of foreign specialists, including Americans who spent vacations, holidays, sabbaticals working in that particular department and in others, to improve the health of the local communities. We have now realised that they also wanted to pick up skills, including surgical skills from Dr Ward and her colleagues ( Stafford. 2016; Kelly & Duffy. 2013). Negative stories about the skills or professionalism of some overseas doctors also circulated there.

Due to the proximity of consultant staff, on call intern doctors in Medicine would stroll down to our home, at the end of their call where they would converse with my father, giving progress reports of the acutely ill patients. We, as children often overheard these conversations, including many other medical discussions between the specialists as they discussed cases, incidents and treatments. From these discussions, one could sense the passion and enthusiasm with which the staff conducted their business- holding their patients in high regard and looking out for their best interests. They used limited resources at the disposal equitably and went over and beyond the call of duty to care. For them it was a vocation.

Though still a child at the time, and now many years later, I continue to be struck by the infectious passion, dedication and professionalism of the healthcare professionals that worked in those settings. Not only had the sisters left their home countries and travelled to Africa but some of them had taken up medical training to equip themselves for their vocation. Their work saved countless lives and the recipient communities in turn loved, respected and protected them. Eventually, the Medical Missionaries of Mary founded other hospitals in the wider area, the nearest of which was about an hour’s car journey, replicating their excellent efforts.

The enduring effects of this early exposure were that we, as children were positively drawn to the healthcare professions. We learned to love and respect the health professions. We could see that it had enormous potential to save lives and heal communities. We began to understand and appreciate the professionalism of its staff without which the foundations of the future would have been unsteady. We could also see first-hand that it called for enormous self-sacrifice and understood that delivery of high quality service called for commitment.

## Tangible Teaching

So there were lots of health professions teaching activities going on at site. The various specialities of nursing could often be seen learning practically outdoors, in groups. The medics had formal and informal departmental teaching meetings. Years later when I went to medical school, my medical class also frequently resorted to peer led study groups and near peer teaching to get through exams. I worked hard to encourage these in junior classes, starting a histology study group which eventually became a class, to help my friends to pass Anatomy.

I often describe my medical school as difficult to get into and difficult to get out of. Medical students lived in trepidation of anatomy exams because students often owed the department marks; scoring - 20, - 60 in anatomy tests. Anatomy comprised Gross Anatomy, Histology and Embryology. I reasoned that if students scored very highly in the histology and embryology components, they were likely to offset the effects of negative marking in gross anatomy. Eventually, going forwards from my class, medical students did form a complex network of informal peer led study groups where a lot of near-teachings were going on. Our pass rates went up and my class, in particular broke all academic records that had previously held in the University’s College of Medicine. Not that I was responsible for these, but I was part of something special at the time. For me, it was a matter of great joy to see my friends and their buddies pass their exams, progressing their careers. The enduring lesson I learned from that period is the enormous power that could be garnered from near peer teaching if put into effective use.

This love for teaching and learning has followed me all my professional life. And from classroom success, it has gone on to the bed side where I am able to clearly see that the quality of clinical and/or bedside teaching has potential to transform a patient’s clinical care and a doctor’s clinical ability.

## Postgraduate Push

The most formidable task and passion of my postgraduate training years had been how to increase the pass rates in the MRCPsych exams among international medical colleagues. This was most acute in the clinical aspects of the exams where advanced communication skills are needed. Here again, having attended various exam preparatory courses and subsequently passed my membership, I set out organising study groups for trainees in my area using simulation techniques, near peer teaching, mentoring and coaching. I can happily say that every trainee that I have been involved in passed their exams and has gone on to be a specialist.

However, doing this for at least four years, I have observed that the cultural background of international doctors affects their communication styles (Pilotto et al. 2007; Verma et al. 2016). Those from the Asian subcontinent tend to be more adept at non-verbal communication skills, noticing and reading body language better than those from the African continent. One may say that the former tend to be more emotionally tuned in. While those from the African continent tend to be better verbal and written communicators. The foundations of these differences tend to be cultural and they can bring different strengths and weaknesses. Some international medical graduates use English language as a second or third language. Few do not think in English. I have spent considerable time, trying to make international learners recognise these differences and make necessary adjustments. It is often not easy to change habits acquired in childhood. This is also applies to issues of professionalism, as notions of what are acceptable professional behaviours have subtle differences that are usually culturally derived ( Odeyale et al. 2017). At times, this teachings happen more in the context of mentorship relationships as adults take time to change behaviours even after they have become aware of them.

## Current Challenges

I have also learned that preparation is pivotal to excellence in clinical teaching. Clinical teaching may be different from academic teaching in various ways. In other for my junior doctor to learn about say, clinical depression, I would have to look out for a patient who may be suited to explain his or her symptoms succinctly and be willing to be interviewed or observed by a junior doctor. This would be followed up by a discussion or series of discussions along with guidance on appropriate reading or study materials including electronic ones to support learning and further time, even in the future, for questions and feedback. The focus is frequently on how the clinical topic is tested for in examinations. International junior doctors may require further adjustments in teaching, to help them assimilate the cultural nuances of symptoms. These implies that clinics or ward rounds and at times job plans have to be structured to make clinical teaching possible and productive. However, this is often not priority for managers including medical managers, as numbers of patients seen and treated tends to be the benchmark for efficiency. Whilst this may be appropriate in itself and in some circumstances; in the current climate of shortage in manpower, teaching time is often squeezed and at times wholly taken up. It can be a battle to protect time to teach juniors.

Those of us who have been longer in the United Kingdom’s National Health Service (NHS) observe that there seems to be less teaching and learning going on. Doctors may be passing exams, depending at times, controversially on their ethnicity and/or nationality ( Wakeford et al. 2015) but readiness and confidence for consultant and/or independent practice seems to be slipping. Junior doctors are complaining of the burden of onerous workplace-based assessments ( Pentlow et al. 2015) and the amount of form filling involved in annual reviews of competency and progression (ARCP) ( Viney et al. 2017). The latter is an annual assessment exercise conducted by training bodies to determine whether a training doctor has satisfactorily met the training requirements for each academic year. It is now to the extent at the workplace that I fear we may be teaching junior doctors to hate medical education.

The fact that junior doctor morale is low is not news ( Goddard. 2016). What is, is the lack of concerted efforts to tackle this. This can only further escalate retention issues. We do not have the numbers yet, but anecdotal information suggests that growing numbers of junior doctors and early career consultants are leaving the medical profession. Motivations may differ but declining work conditions are potent morale killers ( Baldwin et al. 1997). There is a role for clinical educators to stem the tide ( Oliver. 2017), for examples, reduction of escalation in documentation needed to evidence competencies in portfolios and annual appraisals, reducing the burden of endless mandatory training and induction activities, supporting international medical graduates to adjust to their new practice settings. At times, it may just be by way of speaking the truth to educational authorities. I still remember the days when junior doctors worked with a stable multidisciplinary “firm” that grew to know them well, including their strengths and vulnerabilities.

## Conclusion

In summary, international medical graduates may be passionate about their professional callings, however in accessing postgraduate medical education, they face challenges of acculturation and assimilation. These do not occur in a vacuum. These are occurring in an environment (in the UK) of reduced time to teach, increased pressure to document competency attainment and low morale.

## Take Home Messages

• The training needs of international graduates may differ from those of home graduates.
• Clinical teachers need to support international clinical students to become aware and make necessary adjustments in areas such as communication skills and professionalism.
• Near peer teaching has potential to transform learning for international medical graduates.
• Excellence in clinical teaching demands preparation and time, to guide learners.
• Clinical educators have a role to play in improving junior doctor morale and enhancing retention.

## Notes On Contributors

Dr. Itoro Udo MRCPsych MSc is a Consultant Psychiatrist (Locum), with special interest in Medical Education. He holds an MSc in medical education from Durham University. He works at Early Intervention in Psychosis Services, St. John’s Unit, Widnes.

## Declarations

Author can confirm that he is a not a member or a financial supporter of the MMM, however he admires the work of MMM as well as other efforts that improve the lives of less privileged persons.
