# Supplementary material for: There is a Place for Passion
Source: MedEdPublish (2016). 2017 Nov 28;6:213. [Version 1] doi: 10.15694/mep.2017.000213 (PMC10885227; doi:10.15694/mep.2017.000213)
Supplement: Peer review history

# Reviewer report: Reviewer response for version 1

**DOI:** 10.21956/mep.19400.r28454

**Published:** 2017-12-07

**Authors:** P Ravi Shankar

**Affiliations:** 1 American International Medical University

This review has been migrated. The reviewer awarded 4 stars out of 5 I enjoyed reading this well-written paper. The author describes his journey well. I had worked at a missionary hospital for a few months so I am able to place myself in the setting described. The author’s description also reminded me of the United Missions to Nepal (UMN) hospital at Tansen, Palpa, Nepal. Peer and near-peer teaching are very valuable in medical education. I strongly agree with the author that professionalism can be influenced by culture. I also agree that number of patients treated is becoming an increasingly employed parameter of efficiency and protected time allowed for teaching is becoming limited. I was lucky that my teachers during medical school were always willing to structure time for teaching into their daily routine. Documentation is becoming increasingly important and while important it does take up a lot of time. Also a previous paper had examined how the use of OSCE as the predominant method of assessment is increasingly promoting a set of standard behaviors among trainees. These behaviors and practices are usually assessed and learning to concentrate on these may ensure a high score during the OSCE. This paper will be of interest to all health educators.

Reviewer Expertise:

NA

No decision status is available

# Reviewer report: Reviewer response for version 1

**DOI:** 10.21956/mep.19400.r28455

**Published:** 2017-11-29

**Authors:** Neel Sharma

**Affiliations:** 1 Albert Einstein College of Medicine, Montefiore Medical Center

This review has been migrated. The reviewer awarded 5 stars out of 5 I thoroughly enjoyed reading this journey! I firmly believe that by sharing our personal experiences we can better understand the field of education, its issues and solutions. The challenges you mention in the UK are also challenges for trainees in other healthcare systems such as the US and Asia. There has been a push towards competency evidence which has merit but there are certainly some concerns with the over prescriptive nature. Competency should be not only for doctors in training but also for seniors particularly as new drugs and diagnostics are utilised and patient management alters. Seniors are appraised but not in the same way. Typically their competency is assumed.

Reviewer Expertise:

NA

No decision status is available
